# Case of a Student Who Swallowed a Golden Breast-Pin Four Inches in Length, and Voided It by Stool, without Any Ill Consequence

**Published:** 1783

**Authors:** 


IV. *Caſe of a ſtudent who ſwallowed a golden breaſt-pin four inches in length, and voided it by ſtool, without any ill conſequence. Extracted from a letter addreſſed to Dr.* Simmons, F. R. S. *by Mr.* George Bew, *Surgeon; Secretary to the Literary and Philoſophical Society at Mancheſter.*

A Circumſtance has juſt now been communicated to me by one of the teachers of the Warrington Academy, which ſhews in an extraordinary manner the accommodating powers of the inteſtines. A young gentleman, a ſtudent there, holding a golden breaſt-pin in his mouth, whilſt he was changing his ſhirt, had a convulſive ſpaſm of his throat, which he was ſubject to, and ſwallowed it. The pin was between three and four inches in length, and had a head of worked worked hair inclofed in a chryftal oval about the fize of a fixpence. It ftuck in his throat until fome perfon who was near him had laid hold of the point, but a fecond fpafm rendered his attempt to withdraw it impoffible, and it found its way into the ftomach. His fituation was truly alarming, and himfelf and friends had the greateft apprehenfions for his life. The event, however, proved favourable. He was fenfible of its being in his ftomach for a day or two; he afterwards felt the point in various parts of the belly, and at the end of fix days voided it *ex ano*; and, what is very aftonifhing, without its being bent.

*Manchefter,*

